# Reciprocal cross infection of sticklebacks with the diphyllobothriidean cestode *Schistocephalus solidus* reveals consistent population differences in parasite growth and host resistance

**DOI:** 10.1186/s13071-016-1419-3

**Published:** 2016-03-08

**Authors:** Martin Kalbe, Christophe Eizaguirre, Jörn P. Scharsack, Per J. Jakobsen

**Affiliations:** Department of Evolutionary Ecology, Max Planck Institute for Evolutionary Biology, August-Thienemann-Str. 2, 24306 Plön, Germany; School of Biological and Chemical Sciences, Queen Mary University of London, Mile End Road, London, E1 4NS UK; Department of Animal Evolutionary Ecology, Institute for Evolution and Biodiversity, University of Münster, Hüfferstr. 1, 48149 Münster, Germany; Institute for Biology, University of Bergen, Thor Møhlensgt. 55, 5020 Bergen, Norway

**Keywords:** Host-parasite coevolution, Local adaptation, Optimal virulence, *Schistocephalus solidus*, Infection phenotype, *Gasterosteus aculeatus*, Experimental infection

## Abstract

**Background:**

In host-parasite evolutionary arms races, parasites are generally expected to adapt more rapidly, due to their large population sizes and short generation times. There exist systems, though, where parasites cannot outpace their hosts because of similar generation times in both antagonists. In those cases concomitant adaptation is expected.

**Methods:**

We tested this hypothesis in the three-spined stickleback-*Schistocephalus solidus* tapeworm system, where generation times are comparable in both organisms. We chose two populations of sticklebacks which differ prominently in the prevalence of *S. solidus* and consequently in its level of selective pressure. We performed a full factorial common garden experiment. Particularly, Norwegian (NO) and German (DE) sticklebacks, as well as hybrids between both stickleback populations and in both parental combinations, were exposed each to a single *S. solidus* originating from the same two host populations.

**Results:**

We found the infection phenotype to depend on the host population, the parasite population, but not their interaction. NO-parasites showed higher infectivity than DE-parasites*,* with NO-sticklebacks also being more resistant to DE-parasites than to the sympatric NO-parasite. Reciprocally, DE-hosts were more susceptible to the allopatric NO-parasite while DE-parasites grew less than NO-parasites in all stickleback groups. Despite this asymmetry, the ratio of worm to host weight, an indicator of parasite virulence, was identical in both sympatric combinations, suggesting an optimal virulence as a common outcome of parallel coevolved systems. In hybrid sticklebacks, intermediate infection rates and growth of *S. solidus* from either origin suggests a simple genetic basis of resistance. However, comparison of infection phenotypes in NO-maternal and DE-maternal hybrid sticklebacks indicates local adaptation to the sympatric counterpart in both the host and the parasite.

**Conclusions:**

Host-parasite systems with similar generation time show evidence for concomitant reciprocal adaptation resulting in parasite optimal virulence and host parasite specific resistance.

**Electronic supplementary material:**

The online version of this article (doi:10.1186/s13071-016-1419-3) contains supplementary material, which is available to authorized users.

## Background

Hosts and parasites are engaged in an arms race where the evolution of traits associated with the interaction of both antagonists is the result of a mutually influenced trade-off between virulence and resistance. The continuous co-adaptation must therefore be a consequence of the frequency-dependent nature of the selective forces exerted by the parasites and the counter-adaptation of their hosts. The possible outcome of such a co-evolutionary arms race is that either one side reaches a definable optimum, thus preventing the other side from reaching its optimum, or that both sides may reach a mutual local optimum [1].

The conventional assumption is that parasites, due to their shorter generation time, larger population sizes and higher reproductive outputs, are ahead in this co-evolutionary conflict and are therefore more likely to locally adapt to their hosts [2–4]. Numerous empirical studies have investigated local adaptation using model systems with varying levels of complexity, such as bacteria-phage systems [5–8], plant-pathogen associations [9–11], and animal-parasite interactions [12–20]. While several studies found that parasites performed better in their sympatric hosts, others found hosts being better adapted to the local parasites than to allopatric conspecifics, and some studies found no effect at all.

Nevertheless, meta-analyses have identified common tendencies. Specialist parasites with a narrow range of host species are more likely to be locally adapted [21]. Likewise parasites with higher migration rates/gene flow than their hosts are expected to be locally adapted [22, 23]. High virulence in combination with high prevalence also promotes parasite local adaptation [22, 23]. These factors determined from meta-analyses are in accordance with predictions from theoretical modelling approaches, which additionally emphasise the importance of relative host and parasite gene flow/migration rates [15, 24, 25], but also find relative generation time of parasites to have little influence on local adaptation [15, 24].

The majority of local adaptation studies focus on infectivity, i.e. whether a parasite is capable of infecting a certain host genotype or not. This neglects other important traits in the interplay between hosts and parasites, such as infection intensity or virulence, both likely influenced by population-specific ecological factors [18, 26]. In particular, macroparasites with complex life-cycles require the presence of different intermediate and final hosts as well as suitable conditions for successful transmission between them. Hence, they need to achieve multifaceted adaptations to their local environment in order to ensure encountering and successfully infecting hosts, as well as optimal adjustment of the interactions with their hosts’ variable immune defences.

This is the case of the tapeworm *Schistocephalus solidus* and its specific vertebrate intermediate host, the three-spined stickleback *Gasterosteus aculeatus. Schistocephalus solidus* has an obligatory 3-host life-cycle, with piscivorous birds as definitive hosts. From eggs, released with the faeces, the first free-swimming larvae (coracidia) hatch. They need to be eaten by cyclopoid copepods, the first intermediate hosts. In copepods, they develop into procercoids, the stage infective to three-spined stickleback that consume copepods. In the stickleback, the parasite invades the body cavity where it grows as a plerocercoid for several months until the host is preyed upon by a fish-eating bird, where the parasite matures and reproduces [27, 28].

The *S. solidus*-stickleback system is an interesting model for local adaptation studies in several aspects. First, *S. solidus* is extremely specific for three-spined sticklebacks as intermediate hosts [29–31]. Secondly, it performs almost its complete somatic growth already in the stickleback [28, 32, 33], hereby interacting with the two arms of the vertebrate’s immune system: in a naïve fish the parasite is first exposed to unspecific attacks by cells of the innate immune system e.g. granulocytes and monocytes/macrophages [34, 35], as well as various humoral factors, such as those of the complement system [36]. During the several weeks of development the parasite’s massive growth is at least partially controlled by the adaptive immune system [37, 38], which involves T and B lymphocytes and potentially the formation of specific antibodies. Thirdly, sticklebacks in temperate zones are annual and usually experience only a single infection wave in their lives [39, 40], i.e. only one generation of parasites is infecting a host generation. Furthermore, since no multiplication of *S. solidus* takes place in the stickleback, within-host adaptation is not possible. Hence, it is unlikely that the parasite outpaces the evolutionary responses of the host through short generation times and a huge effective population size.

Due to the high mobility of the definitive bird host, potential higher gene flow/migration rates are expected in *S. solidus* compared to its stickleback host. Because of the very short period in which the worm reproduces though, the migratory potential of the definitive host does not seem to result in a panmictic genetic structure in *S. solidus* populations [41]. All these characteristics, especially the high host-specificity for three-spined sticklebacks and the congruence in generation times, which entails equal pace in a co-evolutionary arms race, are prime prerequisites for local adaptation. Therefore, we predict that sticklebacks and *S. solidus* would show variable but concomitant adaptations under different ecological conditions in different geographic localities.

We performed a reciprocal cross-infection experiment with first generation laboratory-bred hosts and parasites from two populations. The populations differed in the prevalence of *S. solidus*: a lake in western Norway with a high prevalence, where we regarded the tapeworm as a major selective force, and a population in northern Germany with a much lower prevalence and, thus, with a relative low selection pressure exerted by *S. solidus*.

In the fully-crossed, factorial experiment, Norwegian and German sticklebacks were exposed to one infective *S. solidus* larva each of either Norwegian or German origin. In order to account for parental effects in the hosts and their potential consequences for the parasites, we also included six hybrid crosses between the stickleback families having either a Norwegian or a German mother. By comparing fitness-relevant parameters in hosts and parasites, we aimed to determine whether infectivity and virulence are attributable mainly to the tapeworm, the stickleback, or both.

## Methods

### Experimental fish

Three-spined sticklebacks were caught in the Neustädter Binnenwasser, a brackish lagoon of the Baltic Sea in Northern Germany (54°06′40″N, 10°48′50″E), and in the lake Skogseidvatnet in Western Norway (60°14′44″N, 5°55′03″E) in winter. In the laboratory, the fish were stepwise acclimatised from winter conditions (6 °C, 8 h:16 h light:dark photoperiod) to summer conditions (18 °C, 16 h:8 h light:dark photoperiod) and were then used for breeding as described in [42]. Three full-sibships of pure Norwegian (NO) and three pure German (DE) pairings were produced and raised in 16 L tanks with continuous water exchange. Additionally, we bred and raised three hybrid sibships of both parental combinations, NO♀ × DE♂ and DE♀ × NO♂, hereafter referred to as NO-maternal and DE-maternal hybrids, respectively. Initially all fish from one egg clutch were kept in one 16 L tank; at the age of 4 to 6 weeks they were subdivided to achieve a density of 20 to 30 sticklebacks per aquarium. Until the age of six weeks, fish were fed daily with live *Artemia* naupliae. Thereafter, they were fed three times per week with frozen copepods, daphnids and chironomid larvae *ad libitum*. At the age of three months, offspring from two to five egg clutches of the same parental pairs (i.e. full-sibs) were combined and transferred to 190 L tanks in densities of 150 to 300 fish per aquarium. Four weeks prior to the start of the experiment, sticklebacks from three sibships of each of the four categories (NO, DE and their two hybrid combinations) were divided into groups of 10 fish each, which were then housed in 16 L tanks again (N_Total_ = 1200, see Table 1).

**Table 1:** Experimental design matrix

	Stickleback origin	NO			DE			NO♀ × DE♂			DE♀ × NO♂		
	Stickleback family	A	B	C	D	E	F	G	H	I	J	K	L
*S. solidus* origin	*S. solidus* family												
DE	P_1	20	20	20	20	20	20	20	-	-	20	-	-
	P_3	20	20	20	20	20	20	-	20	-	-	20	-
	P_5	20	20	20	20	20	20	-	-	20	-	-	20
	*Total*	*180*			*180*			*60*			*60*		
NO	P_2	20	20	20	20	20	20	20	-	-	20		-
	P_4	20	20	20	20	20	20	-	20	-	-	20	-
	P_6	20	20	20	20	20	20	-	-	20	-	-	20
	*Total*	*180*			*180*			*60*			*60*		
Control	-	20	20	20	20	20	20	20	20	20	20	20	20
	*Total*	*60*			*60*			*60*			*60*		

Reciprocal infection scheme of 1200 sticklebacks from Norwegian (NO), German (DE), NO-maternal hybrid (NO♀ × DE♂) and DE-maternal hybrid (DE♀ × NO♂) families (three families each, labelled by capital letters A through L) exposed to sympatric and allopatric *Schistocephalus solidus* families (three families each)

### Experimental parasites

*Schistocephalus solidus* were obtained from the same populations as the sticklebacks and bred in the laboratory. Worms were dissected out from infected fish and were assigned to size-matched pairs, in order to ensure a high rate of outcrossed offspring [43]. Pairs of worms were bred for six days in an *in vitro* system in which the conditions in a bird’s gut were simulated allowing the parasites reproduce, based on Smyth [44], modified by Wedekind [45]. Afterwards, eggs of the different worm pairs (referred to as parasite sibships) were washed thoroughly with cold tap water and stored at 4 °C until use. In order to obtain coracidia, the larval stage infective for copepods, eggs were incubated for 3 weeks at 20 °C in the dark, before light stimulation for hatching [46].

Laboratory cultured copepods *Macrocyclops albidus* served as experimental first intermediate hosts [47]. For controlled infections, medium sized copepods (adult males and C5 females) were placed individually in 24-well microtiter plates with 1.5 mL tap water and starved for one day, before a single *S. solidus* coracidium was added. Copepods were kept at 18 °C and fed with live *Artemia salina* naupliae and *Paramecium caudatum* three times a week. In the second week after exposure, copepods were screened under the microscope for the presence of *S. solidus* procercoids, before they were used for infection of the sticklebacks 15 days post-infection.

### Stickleback infections and time schedule

Experimental infections were performed on six days within a period of three weeks, using one parasite family per day, alternating between NO- and DE-origin. On each infection day 20 sticklebacks from each of three NO- and three DE-host families were exposed to the parasite, together with 20 fish from one of these pure origin families serving as a control.

Due to lower numbers of hybrid fish available, on each infection day 20 sticklebacks from only one NO-maternal and one DE-maternal hybrid family were exposed, another 20 sticklebacks from the respective hybrid families were used as control groups (see Table 1 for the experimental infection matrix and the resulting sample sizes).

Two days prior to exposure, the sticklebacks were transferred individually to small 1 L tanks where each fish received one copepod containing one infective *S. solidus* procercoid. Control fish were each fed with one uninfected copepod. Two days later, sticklebacks were returned to their original 16 L tanks. The water from each exposure tank was filtered and checked microscopically to confirm the ingestion of the copepods.

### Dissections of experimental fish

Eighty-four days after parasite exposure, sticklebacks were dissected in the same order and schedule in which they had been infected. The fish were killed with an overdose of MS222, weighed (+/− 0.1 mg) and the standard length was measured (+/− 1 mm). The abdominal cavity was opened and the plerocercoids of *S. solidus* recovered, as well as the spleen and the liver of the fish were weighed (+/− 0.1 mg).

As parameters for the parasite infection, we calculated the infection rate (percentage of infected fish from all exposed fish) and the relative weight of the tapeworm [Parasite Index in %; PI = (parasite weight/fish + parasite weight) * 100)] [48]. As an indicator of the nutritional status of the fish [49] we calculated the hepatosomatic index (HSI), and as a proxy of immunological activation, the splenosomatic index (SSI), according to the formula (organ weight/fish weight)*100 [50]. For infected sticklebacks, these calculations were done with the fish weight without worm weight.

### Ethics statement

Animal experiments were approved by the Ministry of Energy, Agriculture, the Environment and Rural Areas of the state of Schleswig-Holstein, Germany (reference number: V 313–72241.123-34).

### Statistical analyses

All statistics were performed in R v3.1.2 [51]. First, we tested the infection rate of NO and DE tapeworms in copepods, in order to rule out that DE tapeworms were better at infecting copepods that had originated from the DE population. For this, a χ^2^ test was conducted.

We then tested whether the infection rate in fish (infected *vs* uninfected) was fish family dependent using binomial logistic regression with fish family as a dependent variable. Since family effects were found, we added this variable as a random factor in the following tests. Similarly, because fish sex affects many parameters, this variable was also added as a random factor in the various models.

In order to test for variation in infection rate in the fish, we performed a mixed effect binomial logistic regression with fish family and sex as random factors and fish origin, parasite origin and their interaction as independent variables.

To evaluate parasite growth, we used the Parasite Index (PI) as dependent variable in linear mixed effect model with fish origin, parasite origin and their interaction as independent variables, also including fish family and sex as random factors.

Lastly, fish weight, standard length, hepatosomatic index (HSI) and splenosomatic index (SSI) were tested using similar linear mixed effect models. The same factors as previously described were used, with the exception that *parasite origin* was nested within *host origin* to keep comparisons meaningful since fish morphometric data differ between the origin populations. When needed, Tukey’s honest significant differences, calculated with the *glht* function from the *multcomp* library, were used as post-hoc tests. *P*-values for the fixed effects of the models were obtained using the *lmer* function from the *lmerTest* library [52].

## Results

Norwegian *S. solidus* had a significantly higher infection rate in the laboratory cultured copepods of German origin than the German tapeworms (51.76 *vs* 44.41 %, *X*^*2*^_1,4343_ = 23.50, *P* < 0.01). Even though significant, the infection rates were of a similar order of magnitude, hence, DE-parasites did not infect a laboratory strain of DE copepods obviously better than NO worms, as might be expected if there was adaptation to local copepod hosts.

Out of 1200 sticklebacks, 69 fish died during the experiment and three exposed sticklebacks were found to be accidentally infected by two worms instead of one. These 72 fish were excluded from the analyses.

### Susceptibility of hosts - infectivity of parasites

**Fig. 1:**
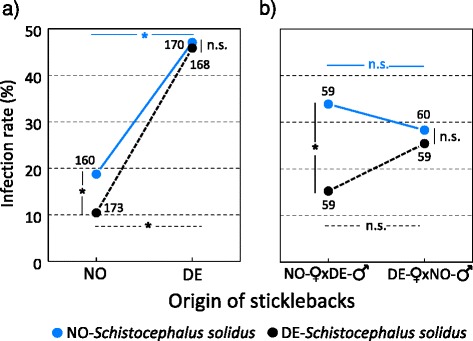
Infectivity of *S. solidus* in different host-parasite combinations. Infection rate (%) in (**a**) pure NO- and pure DE-sticklebacks and (**b**) in F_1_ hybrid sticklebacks of both parental combinations. Each fish was exposed to a single procercoid of *S. solidus* from either the NO- or the DE-population. Significant differences in infection success of *S. solidus* from the two origins within sticklebacks of the same origin (vertical comparison), as well as of *S. solidus* of the same origin between sticklebacks of different origin (horizontal comparison), are indicated by * (*P* < 0.05)

Norwegian sticklebacks were more resistant to infection with *S. solidus*, irrespective of parasite origin, than the pure German sticklebacks (Fig. 1a). While the infection success of the two parasite populations did not differ in pure German sticklebacks, NO-parasites were significantly more infective to pure NO-sticklebacks (Fig. 1a). The infection success of *S. solidus* was intermediate in the stickleback hybrids, suggesting genetic inheritance of resistance to this parasite was additive (Fig. 1b, Tables 2 and 3).

**Table 2:** Statistical results of the various mixed effect models performed

Infection rate			
Variable	*D.F*.	*F*	*P*
Stickleback origin	3	20.290	**<0.001**
Parasite origin	1	5.570	**0.018**
Stickleback origin × Parasite origin	3	3.136	0.277
Relative *S. solidus* growth rate			
Variable	*D.F*.	*F*	*P*
Stickleback origin	3	33.764	**<0.001**
Parasite origin	1	295.371	**<0.001**
Stickleback origin × Parasite origin	3	4.472	**0.004**
Fish standard length			
Variable	*D.F.*	*F*	*P*
Stickleback origin	3	3.930	**0.048**
Stickleback origin × Parasite origin	8	2.118	**0.032**
Fish hepatosomatic index (HSI)			
Variable	*D.F*.	*F*	*P*
Stickleback origin	3	2.158	0.170
Stickleback origin × Parasite origin	8	3.842	**<0.001**
Fish splenosomatic index (SSI)			
Variable	*D.F.*	*F*	*P*
Stickleback origin	3	6.010	**0.016**
Stickleback origin × Parasite origin	8	5.799	**<0.001**

Results are given for infection rate, relative worm growth rate, host standard length, hepatosomatic (HSI) and splenosomatic (SSI) indices, significant *P*-values (<0.05) in bold

**Table 3:** Infection rates of *S. solidus* in the three-spined stickleback

Interaction	Estimate	Standard error	*Z*	*P*
DE-SB *vs* NO-SB	0.287	0.053	5.380	**<0.001**
DE-SB *vs* DE-mat-Hyb	0.134	0.697	1.917	0.217
DE-SB *vs* NO-mat-Hyb	−0.191	0.070	−2.767	**0.028**
DE-mat-Hyb *vs* NO-mat-Hyb	0.058	0.083	0.701	0.895
NO-mat-Hyb *vs* NO-SB	−0.949	0.069	−1.358	0.521
DE-mat-Hyb *vs* NO-SB	−0.153	0.070	−2.179	0.126

Results of a binomial logistic regression; DE denotes the German origin of the stickleback (SB) or the parasite, NO the Norwegian origin, DE-mat-Hyb the hybrid origin of the stickleback with German mother while NO-mat-Hyb refers to the hybrid families with Norwegian mother. Table shows the pairwise comparisons for fish origin of the models described in Table 2, significant *P*-values (<0.05) in bold

The infection rate of the NO-parasite was significantly higher than that of the DE-parasite in the NO-maternal hybrid families, as in the pure NO-sticklebacks(*X*^*2*^_1,120_ = 4.53, *P* = 0.033, Fig. 1b). On the other hand, no difference between the two parasite origins was observed in DE-maternal hybrids and in pure DE-stickleback sibships (*X*^*2*^_1,120_ = 0.17, *P* = 0.680, Fig. 1a and b).

### Parasite growth

**Fig. 2:**
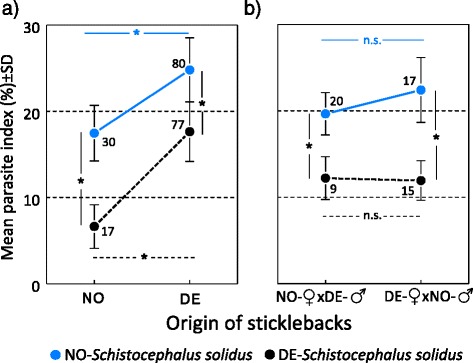
Relative parasite weight in sticklebacks of different origin. Parasite index (%) in (**a**) pure NO- and pure DE-sticklebacks and (**b**) in F_1_ hybrid sticklebacks of both parental combinations. Significant differences in *S. solidus* from the two origins within sticklebacks of the same origin (vertical comparison), as well as of *S. solidus* of the same origin between sticklebacks of different origin (horizontal comparison), are indicated by * (*P* < 0.05)

Investigating the Parasite Index (PI), in all four host groups, NO-*Schistocephalus* grew significantly bigger than DE-worms (mean PI% ± SE; NO-*Schistocephalus* = 29.17 ± 0.534, DE- *Schistocephalus* = 17.891 ± 0.658; *F*_(1,264)_ = 295.371, *P* < 0.001) during the three months of the experiment (Table 2, Fig. 2). Interestingly, the relative worm weight was almost identical in both sympatric host-parasite associations (mean PI% ± SE: 17.332 ± 0.611 in the NO/NO- *vs* 17.616 ± 0.378 in the DE/DE-combination) and showed no statistical difference (*Z* = 0.282, *P* = 0.999, Table 4). The German parasites did not achieve this relative weight in any other fish origin than in the German sticklebacks, whereas the Norwegian worms outperform this level in all but their pure sympatric hosts (mean PI% ± SE: DE-parasite in NO-stickleback 6.866 ± 0.836 *vs* 24.776 ± 0.370 in NO-parasite in DE-Stickleback, Fig. 2a). In both hybrid combinations *S. solidus* from either origin achieved an intermediate size and differences between the two hybrid groups were not significant (Fig. 2b). Nevertheless, there was a maternal effect on the infection phenotype of *S. solidus*: in the DE-maternal hybrids the PI of the NO-parasites differed significantly from that in the pure NO-sticklebacks but not from the pure DE-host. In the NO-maternal hybrids the situation was opposite, where the parasite index of the NO-parasites did not differ significantly from pure NO- but from pure DE-sticklebacks (Table 4). For raw mean values see (Additional file 1: Table S1.

**Table 4:** Relative growth of *S. solidus* in the three-spined stickleback

Fish × Parasite origin	Estimate	Standard error	*Z*	*P*
NO-P + NO-mat-Hyb *vs* NO-P + DE-mat Hyb	−2.723	1.312	−2.075	0.4079
NO-P + NO-SB *vs* NO-P + DE-mat-Hyb	−5.274	1.2451	−4.236	**<0.001**
NO-P + DE-SB *vs* NO-P + DE-mat-Hyb	2.247	1.142	1.968	0.479
DE-P + DE-mat-Hyb *vs* NO-P + DE-mat-Hyb	−10.505	1.159	−9.065	**<0.001**
DE-P + NO-mat-Hyb *vs* NO-P + DE-mat-Hyb	−10.245	1.538	−6.66	**<0.001**
DE-P + NO-SB *vs* NO-P + DE-mat-Hyb	−15.899	1.346	−11.811	**<0.001**
DE-P + DE-SB *vs* NO-P + DE-mat-Hyb	−4.984	1.144	−4.356	**<0.001**
NO-P + NO-SB *vs* NO-P + NO-mat-Hyb	−2.551	1.211	−2.106	0.388
NO-P + DE-SB *vs* NO-P + NO-mat-Hyb	4.970	1.105	4.498	**<0.001**
DE-P + DE-mat-Hyb *vs* NO-PH + NO-mat-Hyb	−7.782	1.345	−5.786	**<0.001**
DE-P + NO-mat-Hyb *vs* NO- P + NO-mat-Hyb	−7.522	1.313	−5.731	**<0.001**
DE-P + NO-SB *vs* NO-P + NO-mat-Hyb	−13.176	1.315	−10.02	**<0.001**
DE-P + DE-SB *vs* NO-P + NO-mat-Hyb	−2.261	1.107	−2.042	0.430
NO-P + DE-SB *vs* NO-P + NO-SB	7.521	1.025	7.340	**<0.001**
DE-P + DE-mat-Hyb *vs* NO-P + NO-SB	−5.231	1.280	−4.087	**0.001**
DE-P + NO-mat-Hyb *vs* NO-P + NO-SB	−4.971	1.453	−3.420	**0.013**
DE-P + NO-SB *vs* NO-P + NO-SB	−10.625	1.010	−10.520	**<0.001**
DE-P + DE-SB *vs* NO-P + NO-SB	0.290	1.027	0.282	0.999
DE-P + DE-mat-Hyb *vs* NO-P + DE-SB	−12.752	1.180	−10.810	**<0.001**
DE-P + NO-mat-Hyb *vs* NO-P + DE-SB	−12.492	1.366	−9.146	**<0.001**
DE-P + NO-SB *vs* NO-P + DE-SB	−18.146	1.145	−15.844	**<0.001**
DE-P + DE-SB *vs* NO-P + DE-SB	−7.231	0.523	−13.828	**<0.001**
DE-P + NO-mat-Hyb *vs* DE-P + DE-mat-Hyb	0.260	1.566	0.166	1
DE-P + NO-SB *vs* DE-P + DE-mat-Hyb	−5.394	1.378	−3.914	**0.002**
DE-P + DE-SB *vs* DE-P + DE-mat-Hyb	5.520	1.182	4.671	**<0.001**
DE-P + NO-SB *vs* DE-P + NO-mat-Hyb	−5.654	1.541	−3.67	**0.005**
DE-P + DE-SB *vs* DE-P + NO-mat-Hyb	5.2604	1.368	3.846	**0.003**
DE-P + DE-SB *vs* DE-P + NO-SB	10.915	1.148	9.511	**<0.001**

Linear mixed effect model on the parasite index. Table shows the pairwise results for the significant interaction between stickleback (SB) origin and parasite (P) origin. For abbreviated parasite and host identities see legend Table 3, significant *P*-values (<0.05) in bold

### Physiological impact on hosts

Stickleback length varied with their origin (*F*_(3,1128)_ = 3.930, *P* = 0.048) and an interaction between fish and parasite origin (*F*_(8,1128)_ = 2.118, *P* = 0.032). No significant pairwise comparison remained after correcting for multiple testing (Table 2).

**Fig. 3:**
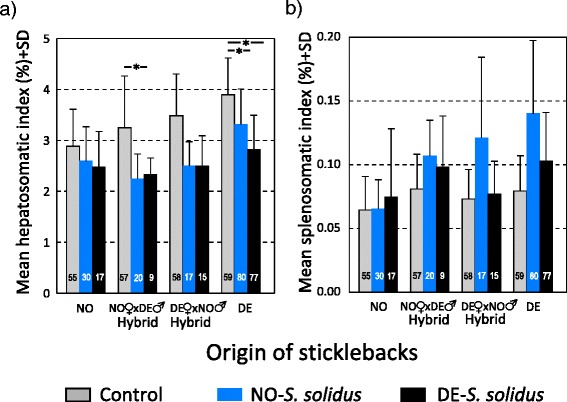
Organ weights in sticklebacks of different origin and treatment groups. Relative weight of (**a**) the liver (hepatosomatic index) and (**b**) the spleen (splenosomatic index), calculated on the basis of total fish weight without weight of the parasite. Significant differences between treatment groups within each stickleback origin are indicated by * (*P* < 0.05)

Fish hepatosomatic index (HSI), which is a proxy for the metabolic reserves stored, did not vary with fish origin (*F*_(3,1128)_ = 2.158, *P* = 0.170), but we detected a significant interaction between fish origin and parasite origin (*F*_(8,1128)_ = 3.842, *P* < 0.001). This interaction was mainly driven by differences observed in DE-fish, where the hepatosomatic index of infected fish was significantly smaller than that of control fish (Fig. 3a). In none of the fish groups were there differences in HSI between infections with the DE- or the NO-parasite (Table 5). Furthermore, we found that NO-maternal hybrid sticklebacks suffered a significantly decreased HSI when infected with a NO parasite compared to control fish (Table 5).For raw mean values see (Additional file 1: Table S1.

**Table 5:** Length and relative size of liver and spleen in experimental sticklebacks

Fish Standard Length (SL)	Estimate	Standard error	*Z*	*P*
NO-P + DE-mat-Hyb *vs* Contr DE-mat-Hyb	−0.173	0.383	−0.451	1.000
DE-P + DE-mat-Hyb *vs* Contr DE-mat-Hyb	0.469	0.383	1.224	0.976
DE-P_ + DE-mat-Hyb *vs* NO-P + DE-mat-Hyb	0.642	0.383	1.675	0.815
NO-P + NO-mat-Hyb *vs* Contr NO-mat-Hyb	0.254	0.390	0.653	1.000
DE-P + NO-mat-Hyb *vs* Contr NO-mat-Hyb	0.306	0.389	0.789	1.000
DE-P + NO-mat-Hyb *vs* NO-P + NO-mat-Hyb	0.052	0.385	0.135	1.000
DE-P + NO-SB *vs* NO-P + NO-SB	−0.621	0.233	−2.661	0.172
NO-P + NO-SB *vs* Contr NO-SB	0.462	0.328	1.407	0.935
DE-P + NO-SB *vs* Contr NO-SB	−0.159	0.327	−0.487	1.000
NO-P + DE-SB *vs* Contr DE-SB	−0.007	0.317	−0.023	1.000
DE-P + DE-SB *vs* Contr DE-SB	0.511	0.318	1.610	0.851
DE-P + NO-SB *vs* NO-P + DE-SB	0.519	0.228	2.275	0.386
Hepatosomatic Index (HSI)	Estimate	Standard error	*Z*	*P*
DE-P + NO-mat-Hyb *vs* NO-P + NO-mat-Hyb	0.153	0.157	0.974	0.996
NO-P + NO-mat-Hyb *vs* Contr NO-mat-Hyb	−0.531	0.160	−3.330	**0.026**
DE-P + NO-mat-Hyb *vs* Contr NO-mat-Hyb	−0.378	0.159	−2.377	0.322
NO-P + NO-SB *vs* Contr NO-SB	−0.148	0.134	−1.099	0.990
DE-P + NO-SB *vs* Contr NO-SB	−0.095	0.134	−0.713	1.000
DE-P + NO-SB *vs* NO-P + NO-SB	0.052	0.096	0.547	1.000
NO-P + DE-mat-Hyb *vs* Contr DE-mat-Hyb	−0.477	0.157	−3.041	0.064
DE-P + DE-mat-Hyb *vs* Contr DE-mat-Hyb	−0.289	0.157	−1.846	0.706
DE-P + DE-mat-Hyb *vs* NO-P + DE-mat-Hyb	0.187	0.157	1.194	0.980
DE-P + DE-SB *vs* NO-P + DE-SB	−0.076	0.093	−0.818	0.999
NO-P + DE-SB *vs* Contr DE-SB	−0.576	0.130	−4.436	**<0.001**
DE-P + DE-SB *vs* Contr DE-SB	−0.652	0.130	−5.015	**<0.001**
Splenosomatic Index (SSI)	Estimate	Standard error	*Z*	*P*
NO-P + NO-SB *vs* Contr NO-SB	−0.010	0.005	−1.878	0.718
DE-P + NO-SB *vs* Contr NO-SB	−0.008	0.005	−1.517	0.912
DE-P + NO-SB *vs* NO_P + NO-SB	0.002	0.004	0.515	1.000
NO-P + DE-SB *vs* Contr DE-SB	0.014	0.005	2.804	0.143
DE-P + DE-SB *vs* Contr DE-SB	0.008	0.005	1.541	0.903
DE-P + DE-SB *vs* NO-P + DE-SB	−0.006	0.004	−1.753	0.799
DE-P + DE-mat-Hyb *vs* NO-P + DE-mat-Hyb	0.182	0.006	−2.966	0.094
NO-P + DE-mat-Hyb *vs* Contr DE-mat-Hyb	0.005	0.006	0.737	1.000
DE-P + DE-mat-Hyb *vs* Contr DE-mat-Hyb	−0.014	0.006	−2.230	0.462
NO-P + NO-mat-Hyb *vs* Contr NO-mat-Hyb	−0.001	0.006	−0.138	1.000
DE-P + NO-mat-Hyb *vs* Contr NO-mat-Hyb	−0.004	0.006	−0.632	1.000
DE-P + NO-mat-Hyb *vs* NO-P + NO-mat-Hyb	0.000	0.006	−0.499	1.000

Linear mixed effect model for the standard fish length (SL), hepatosomatic index (his) and splenosomatic index (SSI) as diagnostic tool of the physiological status of the three-spined stickleback when infected with *S. solidus*. For abbreviated parasite and host identities see legend to Table 3. Contr refers to sham-exposed control sticklebacks. Tables show post-hoc tests for focusing on the significant interaction between fish and parasite origins. Within fish group comparisons are shown, significant *P*-values (<0.05) in bold

The splenosomatic index (SSI), as a proxy for immunological activity, represents the effort of the host to counteract effects of the parasite. Here, we found a significant effect of fish origin (*F*_(3,1128)_ = 6.010, *P* = 0.016) demonstrating the difference in response to infection (Fig. 3b). Furthermore even though we found a significant interaction between host and parasite origin (*F*_(8,1128)_ = 5.799, *P* < 0.001), none of the pairwise comparison, within the fish origin, remained significant after correction for multiple testing (Table 5).

## Discussion

Local adaptation in host-parasite interactions is best seen as higher fitness of each entity in its own habitat [4]. The reciprocal fitness effects a host and a macroparasite exert on each other are rarely so direct and more obvious than that of the sticklebacks - *Schistocephalus solidus* association. Uninfected sticklebacks have a higher fecundity than infected ones, in which the likelihood of reproduction decreases with increasing worm size [40, 53, 54]. Conversely, when *S. solidus* does not successfully infect a stickleback its reproductive success is null, but the bigger it grows in the stickleback, the more offspring it can produce after transmission to its definitive host [32]. This strong reciprocal selective pressure, the high level of host-specificity [29–31] and the long developmental phase in the stickleback [28, 32, 33, 55] are thought to be based on specific genotype-genotype interactions between hosts and parasites and are presumably the reason why allopatric combinations differ so clearly in their infection phenotypes.

### Infection rate and parasite growth

In this full factorial experiment, the Norwegian (NO) sticklebacks were more resistant to infection with both sympatric and allopatric *S. solidus* than all other host groups. The highest infection rate was found in pure German (DE) fish, whereas the hybrids of both parental combinations where at intermediate levels. Like in pure NO-sticklebacks, we found a difference in infection success between NO- and DE-parasites only in the NO-maternal hybrids, which suggests a maternal effect in the F1 hosts. NO-sticklebacks seem to maternally inherit higher susceptibility to their sympatric parasite, while overall being more resistant to *S. solidus* infections. This is likely because NO-parasites are adapted to immunological characteristics of their sympatric hosts, at least partially maternally inherited. These host traits are necessarily compounds of the innate immune system which prevents the initial establishment of the parasite, as clearing an infection may occur only within the first two weeks [35]. In cell culture assays, isolated leucocytes of NO-sticklebacks responded with stronger respiratory burst to *S. solidus* antigens than leucocytes of DE-sticklebacks. In parallel, for both host origins, antigens from NO-parasites elicited higher reactions than those from DE-tapeworms [35].

At the end of our 12-week experiment, *S. solidus* should have almost reached its growth plateau [35, 56]. Parasite size is mainly controlled by the stickleback’s adaptive immune system [37]. The huge differences in the relative parasite weight between NO- and DE-tapeworms, however, clearly show that the growing capacity of *S. solidus* is also a parasite population-specific trait. Since it is negatively correlated with host fitness [40, 53, 57, 58], size of an individual worm is a good proxy for its virulence [59]. Similar to infectivity, NO-parasites grew always better (i.e. were more virulent) than DE-worms in all host groups. At the same time, NO-sticklebacks best controlled the growth of parasites of both origins, which reached their biggest size in the pure DE-fish. In hybrid sticklebacks, tapeworms from both origins reached an intermediate level of growth. There was, however, indirect evidence for maternally inherited differences in immunocompetence in case of interaction with the NO-parasite: in DE-maternal hybrids, parasites grew bigger than in pure NO-sticklebacks whereas there was no significant difference in worm size in comparison with pure DE-hosts. Conversely, the parasite index of NO-parasites in NO-maternal hybrids resembled the index in pure NO-sticklebacks. This also suggests a maternal effect for the ability of the immune system to control parasite growth in the hybrid fish.

We did not test the experimental fish for their Major Histocompatibility Complex (MHC) genotype, a key component of the adaptive immune system which plays a role in controlling *S. solidus* growth [37]. Yet, because MHC variants are bi-parentally inherited we can rule out that differences between the two hybrid combinations are solely due to population-specific MHC-genotypes. Other adaptive host-factors (whether genetic or not) must therefore contribute to the different infection phenotypes, in addition to the apparent intrinsic virulence of the two parasite origins.

### Fitness relevant effects on the hosts

The relative weight of the liver is commonly used as an indicator for the nutritional status of the fish hosts [49, 50]. In the NO-sticklebacks neither of the two tapeworm origins had any significant effect on the hepatosomatic index (HSI), substantiating their ability to cope with the infection. Infected DE-sticklebacks had a lower HSI than uninfected control DE-fish, whereas (possibly due to a smaller sample size) in hybrids only in the DE-maternal combination a significant difference between NO-infected and control sticklebacks was seen. Interestingly, although NO-parasites were always much bigger, there was no difference in the effect of the two parasite origins on the HSI in any of the stickleback origins. This suggests that an infection with the less virulent DE-*parasite* may cause additional costs than simply converting host resources into parasite body mass. These could be for instance costly immune responses, causing trade-offs in the host with other resource-demanding traits like reproduction or growth [60, 61].

The relative size of the spleen of infected fish is an indicator for immunological activity [62–64]. Infected fish showed significantly higher SSI than uninfected fish with the two *S. solidus* strains having different effects on host spleen size: only NO-tapeworms caused a significantly elevated splenosomatic index in DE-fish, as well as in DE-maternal hybrids. This demonstrates the strong immune need to fight these parasites off and further suggests population specific maternally-inherited immune traits. The NO-parasite grew bigger even though it caused higher activation of the hosts’ immune system indicating that the more virulent parasite is less affected by the host’s immunological responses. It is also possible that the host’s immune response itself promotes the increased growth of the parasite through increased uptake of nutrients from the perivisceral fluid *via* parasite’s tegument. For instance, *Ligula intestinalis*, a close relative of *S. solidus*, takes up amino acids from the perivisceral fluid of infected roach [65]. Consequently, the influx of cells and immune mediators into the body cavity may also be beneficial for the tapeworm, as it increases the availability of nutrients and enhance its growth.

### Local adaptation and ecological factors

Our results revealed an asymmetric interaction between fish and their parasites: NO-sticklebacks were more resistant to parasites from both origins and NO-tapeworms grew larger than DE-parasites. The infection phenotypes do not fulfil the ‘local *vs* foreign’ criterion for local adaptation, in which the sympatric parasite performs better than the allopatric one in a given host population [4]. Furthermore, the results also do not meet the ‘home *vs* away’ criterion, where in a direct comparison of host populations, each of the parasite origins would be more successful in the sympatric than in the allopatric host (see Fig. 1 in [4]). What our results suggest instead, is a combination of both host and parasite genotype effects [66], probably without crossing reaction norms [18, 67] where the evolutionary trajectories of the interactions have taken different directions.

A striking result here is the almost identical parasite index in both sympatric host-parasite combinations, which we interpret as an optimal virulence [68–70]. Optimal virulence has been described theoretically [71–74] or tested experimentally in obligatory killing or castrating microparasites [75, 76]. To our knowledge, such a case has not been reported for macroparasites, even though in these parasites there is also an expected trade-off between virulence and the probability of successful transmission [77, 78]. Our results suggest an intrinsic limitation preventing *S. solidus* from maximally exhausting its sympatric host fish to maintain its own maximised reproductive output. Furthermore, the level of virulence is likely an adaptation to ecological factors, which interfere with successful completion of the parasite’s life-cycle. The effects of *S. solidus* on sticklebacks, such as increased conspicuity [79, 80], reduced predator avoidance behaviour [81–84] and escape ability [85], increase the risk of predation not only by birds but also by non-host predatory fish. We therefore uphold the suggestion that highly virulent *S. solidus* are expected to occur where a low density of non-host predator ensures a sufficient transmission rate to piscivorous birds, as supported by exceptional high parasite burdens from water bodies without predatory fish [48, 86] and concluded from the introduction of salmon driving *S. solidus* almost to extinction [87].

Whether or not stickleback populations evolve high levels of parasite resistance may depend on e.g. the parasite abundance that can vary even on a small geographical scale [58, 88, 89]. In the DE-population used here (prevalence < 1 %, M. Kalbe, unpublished), we suggest that the actual risk of becoming infected with *S. solidus* is too low to select for an efficient specific defence strategy.

In contrast, sticklebacks from the NO-population, with a *S. solidus* prevalence ranging between 20 and > 50 % (P.J. Jakobsen, unpublished), face a high risk of becoming infected with a very virulent *S. solidus* and therefore, evolved increased resistance. In response, their sympatric parasites had to evolve more potent immune evasion mechanisms and NO-parasites became more virulent. Conversely, in the German system, the conditions are met for a de-escalating arms race with low selection pressure preventing fish developing a strong immune response, which in turn results in lower parasite virulence levels.

## Conclusions

Overall, the asymmetry in host resistance-parasite virulence described here, indicates concomitant adaptations of co-evolving stickleback-*S. solidus* systems. Conclusions drawn from maternally inherited traits in hybrid sticklebacks suggest that both hosts and parasites show specific adaptations to their sympatric counterparts resulting in population specific coevolutionary trajectories.

## Additional file

Additional file 1: Table S1. Raw mean values for fish standard length and weight, as well as parasite and organs weights of female and male sticklebacks, 12 weeks after infection with *Schistocephalus solidus*. (DOC 71 kb)
