# Supplementary material for: Reciprocal cross infection of sticklebacks with the diphyllobothriidean cestode Schistocephalus solidus reveals consistent population differences in parasite growth and host resistance
Source: Parasit Vectors. 2016 Mar 8;9:130. doi: 10.1186/s13071-016-1419-3 (PMC4782366; doi:10.1186/s13071-016-1419-3)
Supplement: Additional file 1: Table S1. — Raw mean values for fish standard length and weight, as well as parasite and organs weights of female and male sticklebacks, 12 weeks after infection with Schistocephalus solidus. (DOC 71 kb) [file 13071_2016_1419_MOESM1_ESM.doc]

**Reciprocal cross infection of sticklebacks with the diphyllobothriidean cestode *Schistocephalus solidus* reveals consistent population differences in parasite growth and host resistance**

Martin Kalbe^1§^, Christophe Eizaguirre^1,2^, Jörn P Scharsack^1,3^ and Per J Jakobsen^1,4^

^1^Department of Evolutionary Ecology, Max Planck Institute for Evolutionary Biology, August-Thienemann-Str. 2, 24306 Plön, Germany

^2^School of Biological and Chemical Sciences, Queen Mary University of London, Mile End Road, London E1 4NS, UK

^3^Department of Animal Evolutionary Ecology, Institute for Evolution and Biodiversity, University of Münster, Hüfferstr. 1, 48149 Münster, Germany

^4^Institute for Biology, University of Bergen, Thor Møhlensgt. 55, 5020, Bergen, Norway

^§^Correspondence: [kalbe@evolbio.mpg.de](mailto:kalbe@evolbio.mpg.de)

**Additional file 1: Table S1:** Means ±standard deviation of morphological measurements in female and male sticklebacks 12 weeks after experimental infection with *Schistocephalus solidus*. Host groups are Norwegian (NO) and German (DE) sticklebacks (SB), as well as NO-maternal (NOmat) and DE-maternal (DEmat) stickleback hybrids. Treatment groups are sticklebacks infected with either one Norwegian parasite (NO_P), one German parasite (DE_P) or sham-exposed control. Relative parasite weight is given as Parasite Index (parasite weight/(fish weight including parasite weight)*100) as percentage, relative weight of livers and spleens are given as indices expressed as percentage of organ weight of the host weight (organ weight/(fish weight without parasite weight)*100).

| Host Group | Parasite Treatment | Sex | n | Standard Length (mm) | Fish Weight (mg) | Parasite Weight (mg) | Liver Weight (mg) | Spleen Weight (mg) | Parasite Index (%) | Hepato-somatic Index (%) | Spleno-somatic Index (%) |
| --- | --- | --- | --- | --- | --- | --- | --- | --- | --- | --- | --- |
| NO_SB | NO_P | f | 18 | 43.22±2.76 | 882±134 | 160.68±31.36 | 19.73±5.72 | 0.48±0.22 | 18.37±3.24 | 2.72±0.64 | 0.064±0.023 |
|  |  | m | 12 | 43.08±2.44 | 868±129 | 138.96±32.41 | 18.03±6.55 | 0.49±0.21 | 16.11±3.32 | 2.41±0.69 | 0.067±0.023 |
|  | DE_P | f | 6 | 42.17±3.06 | 744±137 | 48.88±22.75 | 19.42±6.52 | 0.57±0.38 | 6.40±2.03 | 2.80±0.92 | 0.088±0.077 |
|  |  | m | 11 | 42.09±1.81 | 748±121 | 51.16±24.90 | 16.19±5.06 | 0.46±0.25 | 6.78±2.85 | 2.30±0.51 | 0.067±0.039 |
|  | Control | f | 22 | 42.55±1.26 | 692±67 |  | 23.07±7.05 | 0.43±0.16 |  | 3.30±0.80 | 0.062±0.024 |
|  |  | m | 33 | 42.15±1.73 | 714±93 |  | 18.73±4.96 | 0.47±0.20 |  | 2.61±0.52 | 0.066±0.028 |
| DE_SB | NO_P | f | 37 | 41.86±2.53 | 919±145 | 216.45±36.15 | 24.98±7.42 | 0.87±0.36 | 23.74±3.23 | 3.53±0.73 | 0.126±0.051 |
|  |  | m | 43 | 39.74±2.01 | 805±98 | 205.61±32.26 | 18.90±5.40 | 0.91±0.40 | 25.72±3.90 | 3.13±0.61 | 0.152±0.060 |
|  | DE_P | f | 35 | 41.63±2.28 | 827±114 | 147.23±31.63 | 19.47±6.84 | 0.65±0.24 | 17.90±3.57 | 2.82±0.73 | 0.095±0.030 |
|  |  | m | 42 | 41.00±2.50 | 809±122 | 141.10±34.81 | 19.06±5.61 | 0.74±0.30 | 17.45±3.37 | 2.84±0.62 | 0.110±0.042 |
|  | Control | f | 36 | 41.17±2.02 | 699±86 |  | 27.63±7.51 | 0.54±0.20 |  | 3.91±0.78 | 0.079±0.031 |
|  |  | m | 23 | 40.04±1.92 | 672±089 |  | 26.26±6.93 | 0.54±0.17 |  | 3.87±0.65 | 0.081±0.022 |
| NOmat_Hyb | NO_P | f | 11 | 42.91±2.81 | 918±108 | 186.94±24.27 | 17.50±4.51 | 0.80±0.25 | 20.45±2.34 | 2.38±0.44 | 0.109±0.028 |
|  |  | m | 9 | 44.33±1.87 | 960±114 | 180.26±36.09 | 16.48±5.50 | 0.81±0.23 | 18.69±2.33 | 2.08±0.52 | 0.105±0.029 |
|  | DE_P | f | 4 | 45.25±3.20 | 854±148 | 109.28±36.15 | 16.00±3.02 | 0.73±0.26 | 12.60±2.46 | 2.16±0.32 | 0.100±0.042 |
|  |  | m | 5 | 44.40±2.07 | 955±88 | 114.78±32.01 | 20.74±0.83 | 0.82±0.40 | 11.92±2.73 | 2.48±0.25 | 0.097±0.044 |
|  | Control | f | 24 | 43.75±2.27 | 816±133 |  | 30.8±13.17 | 0.63±0.23 |  | 3.69±1.18 | 0.076±0.023 |
|  |  | m | 33 | 42.67±1.78 | 776±102 |  | 22.54±5.28 | 0.65±0.24 |  | 2.93±0.75 | 0.085±0.030 |
| DEmat_Hyb | NO_P | f | 5 | 41.40±2.41 | 865±135 | 176.12±22.35 | 17.06±4.22 | 0.70±0.20 | 20.59±2.64 | 2.48±0.47 | 0.103±0.026 |
|  |  | m | 12 | 40.92±3.34 | 820±139 | 187.53±29.55 | 15.88±4.10 | 0.83±0.49 | 23.19±3.98 | 2.51±0.49 | 0.129±0.073 |
|  | DE_P | f | 7 | 43.29±2.93 | 873±169 | 94.20±28.54 | 21.39±9.54 | 0.57±0.16 | 10.63±1.76 | 2.65±0.71 | 0.073±0.013 |
|  |  | m | 8 | 42.25±1.98 | 807±132 | 105.13±20.70 | 16.83±5.13 | 0.55±0.18 | 13.10±2.13 | 2.37±0.48 | 0.081±0.033 |
|  | Control | f | 26 | 41.92±2.76 | 720±130 |  | 27.55±7.92 | 0.52±0.15 |  | 3.81±0.78 | 0.073±0.020 |
|  |  | m | 33 | 41.81±2.35 | 713±117 |  | 23.14±7.62 | 0.53±0.19 |  | 3.21±0.77 | 0.073±0.026 |
